# Prevention of haemoglobin glycation by acetylsalicylic acid (ASA): A new view on old mechanism

**DOI:** 10.1371/journal.pone.0214725

**Published:** 2019-04-15

**Authors:** Shabnam Ghazanfari-Sarabi, Mehran Habibi-Rezaei, Rosheh Eshraghi-Naeeni, Ali Akbar Moosavi-Movahedi

**Affiliations:** 1 School of Biology, College of Science, University of Tehran, Tehran, Iran; 2 Nano-Biomedicine Center of Excellence, Nanoscience and Nanotechnology Research Center, University of Tehran, Tehran, Iran; 3 Institute of Biochemistry and Biophysics, University of Tehran, Tehran, Iran; 4 Center of Excellence in Biothermodynamics, University of Tehran, Tehran, Iran; Shiraz University, ISLAMIC REPUBLIC OF IRAN

## Abstract

Diabetic hyperglycemia provokes glycation of haemoglobin (Hb), an abundant protein in red blood cells (RBCs), by increasing its exposure to carbohydrates. Acetylsalicylic acid (ASA; Aspirin) is one of the first agents, which its antiglycation effect was witnessed. Although the precise molecular mechanism of action of ASA on protein glycation is not indisputably perceived, acetylation as its main molecular mechanism has been proposed. This report aims to unravel the meticulous mechanism of action of ASA by using two ASA analogues; benzoic acid (BA) and para-nitrobenzoic acid (NBA), despite their lack of acetyl group. In this regard, the inhibitory effect of these two chemicals in comparison with ASA on Hb fructation is reported. UV-visible spectroscopy, intrinsic advanced glycation end products (AGE) fluorescence spectroscopy, extrinsic thioflavin T (ThT) binding fluorescence spectroscopy, 2,4,6-trinitrobenzenesulfonic acid (TNBSA) assay, and single cell gel electrophoresis (SCGE) were used to explore the effects of BA and NBA in comparison with aforementioned chemicals in the context of protein glycation. In spite of the lack of acetyl substitution, NBA is reported as a novel agent with prominent inhibitory efficacy than ASA on the protein glycation. This fact brings up a possible new mechanism of action of ASA and reconsiders acetylation as the sole mechanism of inhibition of protein glycation.

## 1 Introduction

Diabetes is a condition in which the concentration of blood carbohydrates, and consequently the range of glycation increase simultaneously, which result in hyperglycemia, oxidative stress and diabetic complications. Glycation was first described by Louis Camille Maillard in 1912 [1], consisting of two stages. At the first stage, carbonyl groups of reducing sugars including aldoses and ketoses reversibly react with free ε-amino groups of lysines and the α-amino groups at the N-terminal of proteins in order to form respectively labile aldimines and ketimines, which are collectively named as Schiff bases. These compounds easily undergo intramolecular rearrangements at the second stage to form relatively stable early glycation products, known as Amadori [2] and Heynes [3] products.

These early-stage glycation products readily undertake irreversible processes such as glycoxidation, dehydration, condensation, cross-linking, cyclization etc to generate yellow-brown colored, fluorescent, highly cross-linked and weakly soluble species known as advanced glycation end products (AGEs) [4]. In this process, the structure and function of proteins are adversely being altered, most importantly, blood proteins with a long half-life, such as haemoglobin (Hb), are more likely to undergo glycation under hyperglycemic condition [5]. Although the blood glucose is 10- to 1,000-fold greater than fructose concentration, fructose tends to accumulate in erythrocytes through the metabolic polyol pathway [6] and transporter-mediated influx involved by a membrane-associated glucose transporter isoform GLUT5 [7]. The mentioned transporter acts particularly on fructose and possesses approximately 10 fold lower *Km* than glucose specialized transporter; GLUT2 [8]. Serum fructose concentration in non-diabetics and diabetics has been reported about 8±1 μmol/L and 12±4 μmol/L respectively using ^13^C6-fructose as an internal standard [6,9]. Accordingly, the amount of fructose in the erythrocytes of diabetic patients has been reported to be 4 times higher than its amount in non-diabetics [10]. Kawasaki *et al*. (2004) reported 8 times more abundance of fructose in its open form than that of the glucose, owing to the fact that glycation potency correlates with the percentage of sugars in their open form [11], it prevails more reactivity over glucose [12]. Therefore, fructose is an important player in the glycation of Hb or fructation. Fructose reacts non-enzymatically with free amino groups of certain lysines and more importantly with the NH_2_-termini of the α- and β-subunits of Hb. This process takes place gradually and ceaselessly during the 120 days of erythrocyte lifespan [13]. Moeover, D-ribose has been recently introduced as a player in HbA1c formation [14]. Nevertheless, D-ribose involvement is debatable and remains to be explored whether it plays an eminent role in the type 2 diabetes mellitus (T2DM) [14]. Hb fructation leads to various structural and functional modifications such as heme loss, which leads to iron releasing and free radical production [15], and consequently, causes α to β conformational transition, enhances protein surface hydrophobicity, alleviates both oxygen affinity and peroxidase activity and conversely increases esterase activity [16,17]. Glycated Hb, also known as HbA1c, is a perceptible diagnostic marker for T2DM screening [18], which gives exceptional ability to examine the glycemic condition during past 2–3 months [18]. Due to the importance of glycation in diabetes and its complications, antiglycation therapies considered as a preventive strategy against the formation of AGEs, generation of free radicals through autoxidation of glucose and glycated proteins, oxidative stress and its consequences in diabetic complications. Although various antiglycation compounds have been studied, none has been approved for clinical usage [19]. In the case of mechanism of anti-glycation effect, some are competitors for binding to protein amino groups, some have the ability of binding to proteins at the glycation sites, some can cut out the acyclic forms of reducing sugars and the others are able to attach to glycation intermediates to inhibit the glycation progress and thus AGEs formation [20]. Acetylsalicylic acid (ASA) or aspirin is one of the first molecules, which its anti-glycation function was documented upon *in vivo* and *in vitro* studies [21]. ASA is considered as non-steroidal anti-inflammatory drugs (NSAIDs), which is able to ease the inflammation, the primary pathogenic insult of diabetes progress, by inhibiting the prostaglandins synthesis and cyclooxygenase-2 (COX2) [22]. Despite proven health benefits, various adverse effects of ASA have been reported including gastrointestinal ulcerations, nephrotoxicity, iron deficiency anemia and occult bleeding related to gastrointestinal erosion [23], blood disorders such as anemia and cytopenias agranulocytosis, leucopenia and hemolytic anemia [24]. Although acetylation mechanism has been suggested to describe the anti-glycation effect of ASA [21], the mechanism of action of ASA has not been well perceived yet. To shed light on the ASA’s molecular mechanism with respect to inhibition of protein glycation and explore new compounds with an inhibitory effect on glycation along with approving efficacy and fewer side effects rather than ASA, we are exploring the anti-glycation effect of two structurally ASA-related candidates including benzoic acid (BA) and *para*-nitrobenzoic acid (NBA).

## 2 Materials and methods

### 2.1 Materials

Bovine hemoglobin (Hb), acetyl salisylic acid (ASA), benzoic acid (BA) and *para*-nitrobenzoic acid (NBA) were obtained from Sigma Chemical Co. Trinitrobenzene Sulfonic Acid (TNBSA) was purchased from Fluka. All other chemicals were purchased from Merck.

### 2.2 Hb treatment

A 15 μM bovine Hb was incubated in the presence of 30 mM fructose in 50 mM phosphate buffer (pH 7.4). Each of BA, NBA, and ASA were added in pursuance of analyzing their protection against glycation process at the final concentration of 1.5 mM [16]. Due to the limitation of using sodium azide as a preservative agent in Hb incubation [25] sterilizing of all of the solutions was performed using low protein binding filter (Millex–GV 0.22 μm filter unit, Millipore). Finally, solutions were incubated at 37°C under sterile conditions. Control samples were prepared in a similar way in the absence of fructose. Sampling was done at appropriate time intervals up to 20 days of incubation, then samples were kept at ‒70°C until analyzing.

### 2.3 Spectroscopic analysis

Soret band absorption, intrinsic Hb-AGE-related, and extrinsic thioflavin T (ThT) binding fluorescence assessments were carried out. Soret band absorption was studied using a Synergy HT Multi-Mode Microplate Reader using a quartz microplate at the final protein concentration of 0.125 μM. Spectrum was recorded in the range of 250–700 nm. Fluorescence analysis was performed using a Varian Cary Eclipse fluorescence spectrophotometer. AGE-dependent fluorescence intensity of samples was recorded in the excitation/emission wavelength pair of 370/450 nm at 25°C. Fluorescence intensity of samples was read against buffer as blank. The final concentration of Hb was 1.5 μM.

ThT, a benzothiazole fluorescent dye, which shows remarkable fluorescence intensity increment after attachment to amyloid aggregates, is the most typical marker for the indication of β-sheets aggregates. This dye is used to assess the fibrillation process and amyloid fibrils genesis *in vitro* at excitation/emission pair of 435/505 nm by fluorescence spectroscopy [26]. In brief, Hb samples in the final concentration of 1.5 μM were analyzed using 1 mg/ml ThT at the final concentration of 5 μM in 50 mM phosphate buffer (pH 6). Before ThT fluorescence assessment, all of the samples were pre-incubated for 10 minutes in room temperature in the dark then the emission of samples was scanned in the wavelength range of 455–455 nm after excitation at 435 nm. All evaluations were done at least thrice and the standard deviations (SD) and averages were used for further analysis.

### 2.4 Free amine content

2,4,6 Trinitrobenzenesulfonic acid assay (TNBSA) was used in order to estimate free amine content of lysine residues in Hb before and after incubation time in the absence and presence of fructose and three desired chemicals including ASA, NBA, and BA, according to Fields *et al*. (1972) [27] with slight modifications. In brief, 0.1 M sodium bicarbonate (pH 8.5) as a reaction buffer and 0.25 ml freshly made 0.01% (W/V) TNBS solution were added to 100 μg/ml protein samples at the volume of 0.5 ml. After incubation at 37°C for 2 hours in the dark, the reaction was stopped by adding 0.25 ml of 10% SDS and 0.125 ml of 1 N HCl and followed by reading the absorbance at 335 nm. Knowing the 48-lysine residues per mole of Hb, the number of modified lysines (τ) corresponds to glycation degree was calculated by the formula of [28]:

$$\tau =\frac{\left(\text{OD control}-\text{OD modified}\right)\times \text{Number of Lysines}}{\text{OD control}}$$

### 2.5 Single cell gel electrophoresis (SCGE) analysis

The evaluation of fructation and chemicals genotoxic features were achieved using single cell gel electrophoresis (SCGE also known as comet assay) under alkaline condition according to the procedures of Singh *et al*. (1988) [29]. In brief, isolated lymphocytes obtained from a healthy blood bank donor, exposed to (almost 35,000 cells per each sample) 50 μg/ml Hb aggregates 15 μM, adjusted to 1 ml by adding sterilized phosphate buffered saline (PBS), followed by incubation for an hour at 37°C in the dark. Then, the reaction solutions were centrifuged at 717 ×g, followed by removing supernatants and resuspending cells by adding 100 μl of sterilized PBS. Afterward, 80 μl of 1.0% low melting point agarose (LMPA, dissolved in PBS1X) was added to each cells suspensions and immediately pipetted over slides, which were pre-coated by 1.0% normal agarose (dissolved in PBS1X) and then covered by coverslips. After storing the slides on the ice patch for 10 minutes, coverslips were removed and the next layer of 0.5% LMPA as a third layer was pipetted over the slides and the same procedure was performed and followed by immersing slides (without coverslips) in the cold lysis solution (2.5 M NaCl, 100 mM EDTA and 10 mM Tris, pH 10). Freshly made 1% Triton X-100 and 10% DMSO were added 30 minutes before using the buffer, protected from light and was kept at 4°C for 12 hours. After two times washing slides by storing them in the cold distilled water for 5 minutes and unwinding the DNA by keeping slides in the alkaline electrophoresis buffer (300 mM NaOH and 1 mM EDTA pH>13), the electrophoresis was carried out at 4°C in the field strength of 20 volts and 300 milliampere for 20 minutes. The slides were then immersed in the cold neutralization solution (0.4 M Tris pH 7.5) for 15 minutes followed by immersing in pure ethanol for 5 minutes and drying in the room temperature [30]. Slides were stained with 1X ethidium bromide and covered by coverslips before analyzing by fluorescent microscope filter 516–560 nm, 590 nm barrier filter, and total magnification of 400X. After recording more than 20 comets for each sample, comets were analyzed by OpenComet 1.3 imageJ application which is available at *www.opencomet.org*.

## 3 Results and discussion

**Fig 1:**
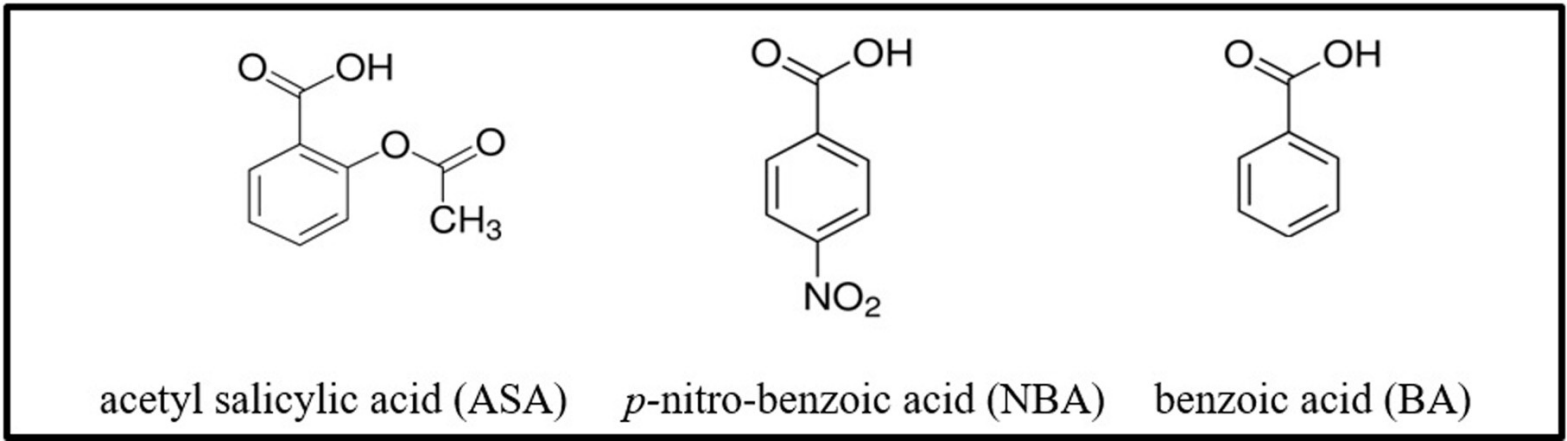
The chemical structures of the ASA, NBA, and BA.

Nowadays, diabetes is a major public health problem featured by hyperglycemia. As the main consequence of diabetic condition, long-lived proteins such as Hb become the main target of glycation, which reveals the complications of diabetes. In diabetics, serum fructose concentration is increased 1.5 fold, meanwhile, such increase in RBCs showed to be 4-fold [9], which consequently introduce fructose as one of the main players of the Hb glycation or fructation. A considerable amount of literature has been published on the inhibitory effect of ASA on protein glycation [16,21,31]. Moreover, there are also some documents dealing with the side effects of ASA consumption [23,24]. Considering this issue, the inhibitory effect of BA as a basic structure and NBA as a derivative of BA on Hb fructation is reported in comparison with ASA (Fig 1).

**Fig 2:**
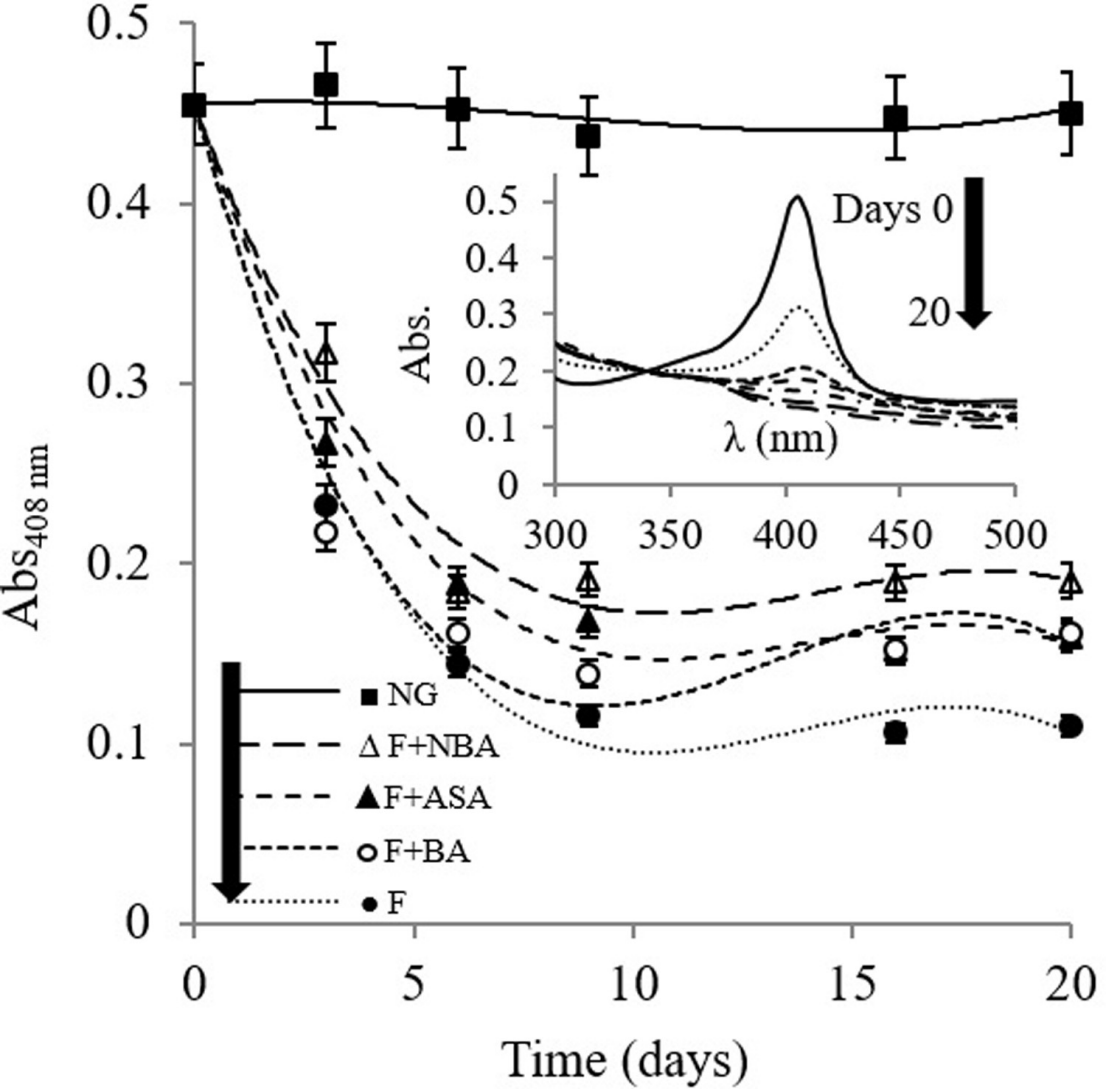
Study of heme degradation by evaluating Soret band absorbance variation at 408 nm. Hb was incubated with (F) and without (NG) fructose,in the presence or absence of each of of ASA, NBA, or BA. The inset to figure shows the Soret band absorbance spectra as the function of incubation time in the presence of fructose and absence of inhibitors.

Fig 2 shows the Soret band absorption alteration upon Hb fructation in the absence or presence of ASA, BA, and NBA. The electronic absorption spectrum of porphyrin ring in Hb shows a representative peak known as Soret or B band ranging around 400 nm in the blue region (400–436 nm). This strong transition is assigned from ground state or S_0_ to the second excited state S_2_ with strong intensity [32]. We have already reported fructose as a stimulator of heme/hemin oxidative degradation during the Hb/metHb glycation in which structural alteration of the fructated and non-fructated Hb species was recorded using near UV-visible spectroscopy [33]. As shown, the absorbance of Hb at Soret region in the absence of fructose as control does not experience significant change during fructation process however, full-fructated Hb exhibited an enormous hypochromic effect in the Soret band region after 20 days of incubation, which is in accordance with previous reports [33,34]. According to the protective role of ASA against glycation [16], while aspirin only reduced 12% of observed hypochromism effect of fructation, NBA and BA inhibited fructation induced hypochromism more effective than ASA and were able to reduce 24 and 15%, respectively (Fig 2 and S1 Table). Fructation would also generate free radicals acting as vital elements in heme degradation [35]. Among them, the moderate steady-state generation of H_2_O_2_ in glycation thought to be responsible for remarkable heme degradation and destructive effect on globin structure [34]. These findings confirm considerable conformational alteration of the heme pocket, which leads heme release from the hydrophobic pocket [36] and results in hypochromocity in the Soret region of the spectrum (Fig 2). NBA revealed more effective inhibition on the structural changes of Hb, and heme group destruction than ASA or BA in the presence of fructose which present NBA as an effective anti-glycation agent.

**Fig 3:**
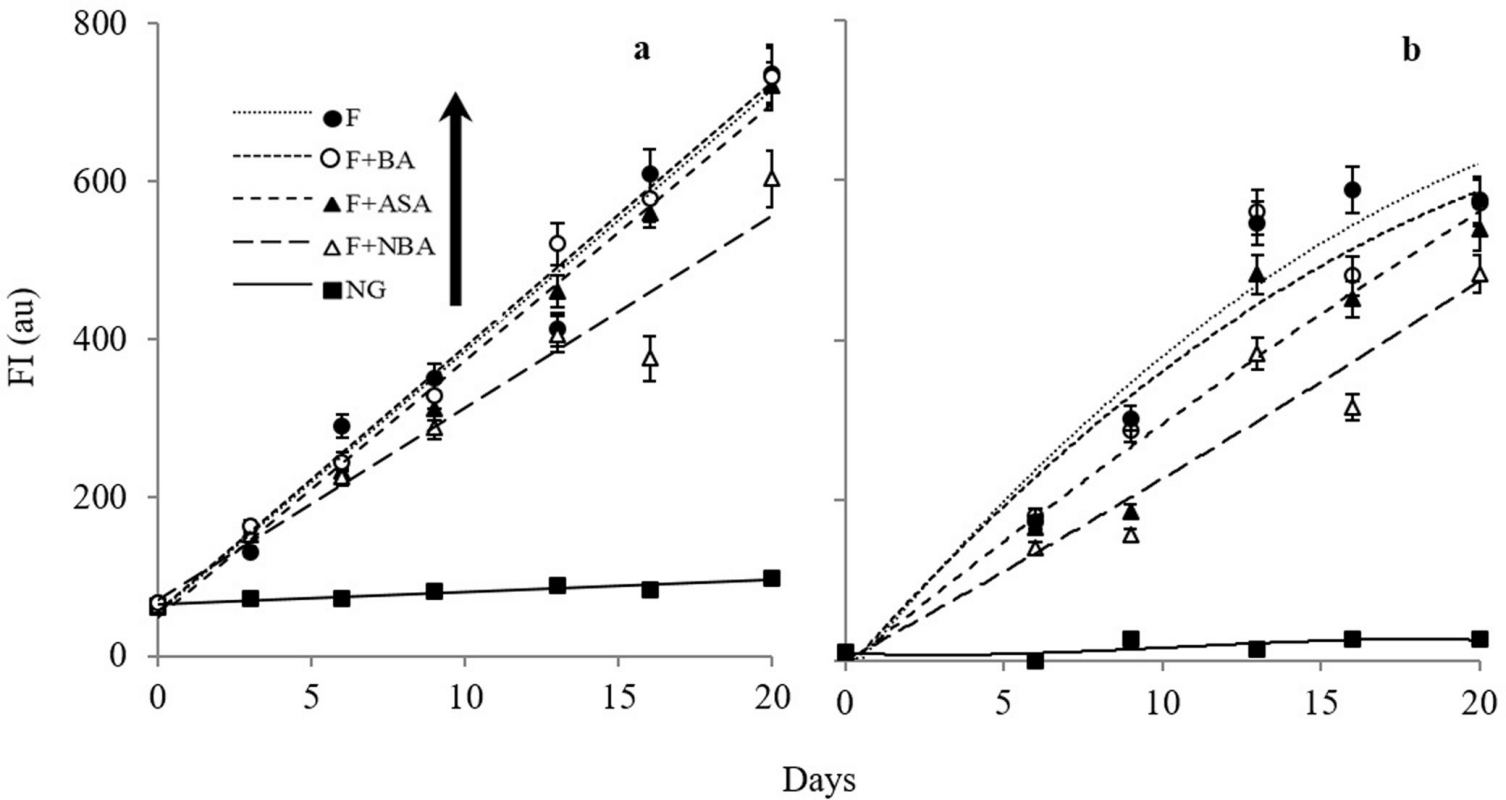
The Inhibitory effects of ASA, NBA and BA on Hb glycation using intrinsic AGE–dependent and extrinsic ThT–dependent fluorescence spectroscopy. (a) 1.5 μM Hb-AGE-dependent fluorescences (λ_ex_ 370, λ_em_ 440) as the function of incubation time (days) were monitored in the absence of fructose (NG) or presence of fructose (F) and each of 1.5 mM ASA, NBA, and BA. Mean values were determined from three independent experiments and The linear regression coefficients (*R*^*2*^ values) were resulted to be 0.95 ≤ for all samples. (b) Extrinsic ThT–dependent fluorescence spectroscopy data as a function of days of fructation are presented in which the emission spectra were recorded at 505 nm after excitation at 435 nm for the 1.5 μM Hb samples in the abscense (NG) or presence of fructose (F). In addition, the ThT dependent fluorescence emissions as the function of time in the presence of 1.5 mM ASA, NBA, and BA are included.

Fig 3 represents the attenuating effect of ASA, BA, and NBA on intrinsic AGE-dependent and extrinsic ThT-dependent fluorescence of Hb in the period of glycation by fructose and the formation of AGE species, among them NBA represented superior attenuation effect. The AGE-dependent fluorescence was investigated by excitation at 370 nm and recording the emission at 440 nm during 20 days of incubation according to the reported fluorescence descriptive pair by Nakamura et al. 1997 [37]. This specific fluorescence wavelength pair (λ_ex_ 370, λ_em_ 440) is considered to be one of the characteristics of AGEs, which is more likely to be generated upon intra- and inter-molecular process including glycoxidation, dehydration, condensation, cross-linking and cyclization [38,39]. As depicted in Fig 3A, although all of the fructated samples showed an increase in intrinsic AGE-dependent fluorescence, a little fluorescence intensity change was observed for incubated Hb in the absence of fructose. Accordingly, NBA represents higher protective effect on the generation of AGE species upon Hb fructation than the fructated samples in the presence of ASA or BA in the period of 20 days (Fig 3A and S2 Table). ThT, a benzothiazole dye used to explore β sheets *in vitro* and *in vivo*, as a general indicator for amyloid fibril formation, known as amyloidogenesis [40]. As illustrated in the Fig 3B, the extrinsic ThT fluorescence emission intensities were monitored for Hb samples incubated in the presence of fructose for a 20 days incubation period in the absence or presence of ASA, BA, and NBA in which excitation/emission at 440/505 nm was used to assess fibrillation [41]. A strong increase in the emission intensity at 505 nm for fructated Hb samples was observed due to the amyloids formation, which is in correspondence with previous findings [41]. As shown in the Fig 3B, in comparison with Hb incubated in the absence of fructose as a control (NG), a strong increase in emission intensity at 505 nm was recorded for fructated Hb samples, however among samples incubated in the presence of each of ASA, BA or NBA, the most preventive effect was in reference to NBA, which proved to be able to protect against fibrillation more effective than ASA or BA (S3 Table).

**Fig 4:**
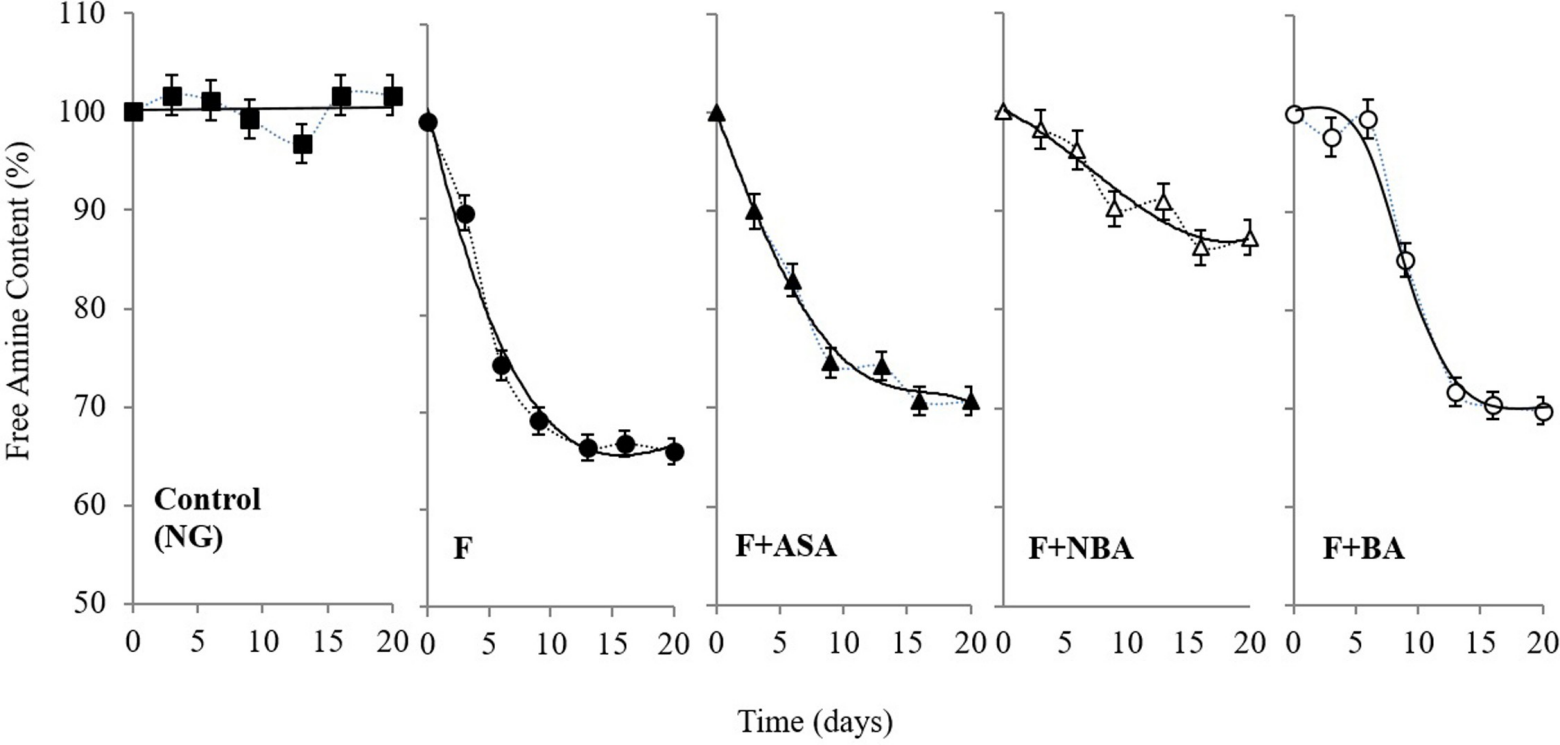
The effect on the percentage of free amine content. The percentage of residual free amine content of Hb as the function of time (in days of incubation) in the absence (NG) as control, presence of fructose (F), in the presence of fructose and each of ASA, NBA and, BA at 37°C in 50 mM phosphate buffer. The results are the average of three independent repeats.

At open ring state, carbonyl groups in carbohydrates are able to non-enzymatically react with free primary amines of protein N-termini and ε-amines of lysine residues during protein glycation [42]. Therefore, determination of free amine contents by applying TNBSA could be an eminent index for glycation progression [43]. According to data presented in Fig 4, lack of fructose caused the constant free amine content during Hb incubation, yet there has been a sharp decrease in the number of free lysine residues in fructated Hb species. However, decreasing of free amine content along with the fructation of Hb in the presence of ASA, BA, and NBA observed to be harnessed, among them NBA was proven again to act more effective by preserving 87.2% of free amine content, than ASA and BA, which remained 70% and 69.7% of free amine content at the end of the incubation (Fig 4 and S4 Table).

**Fig 5:**
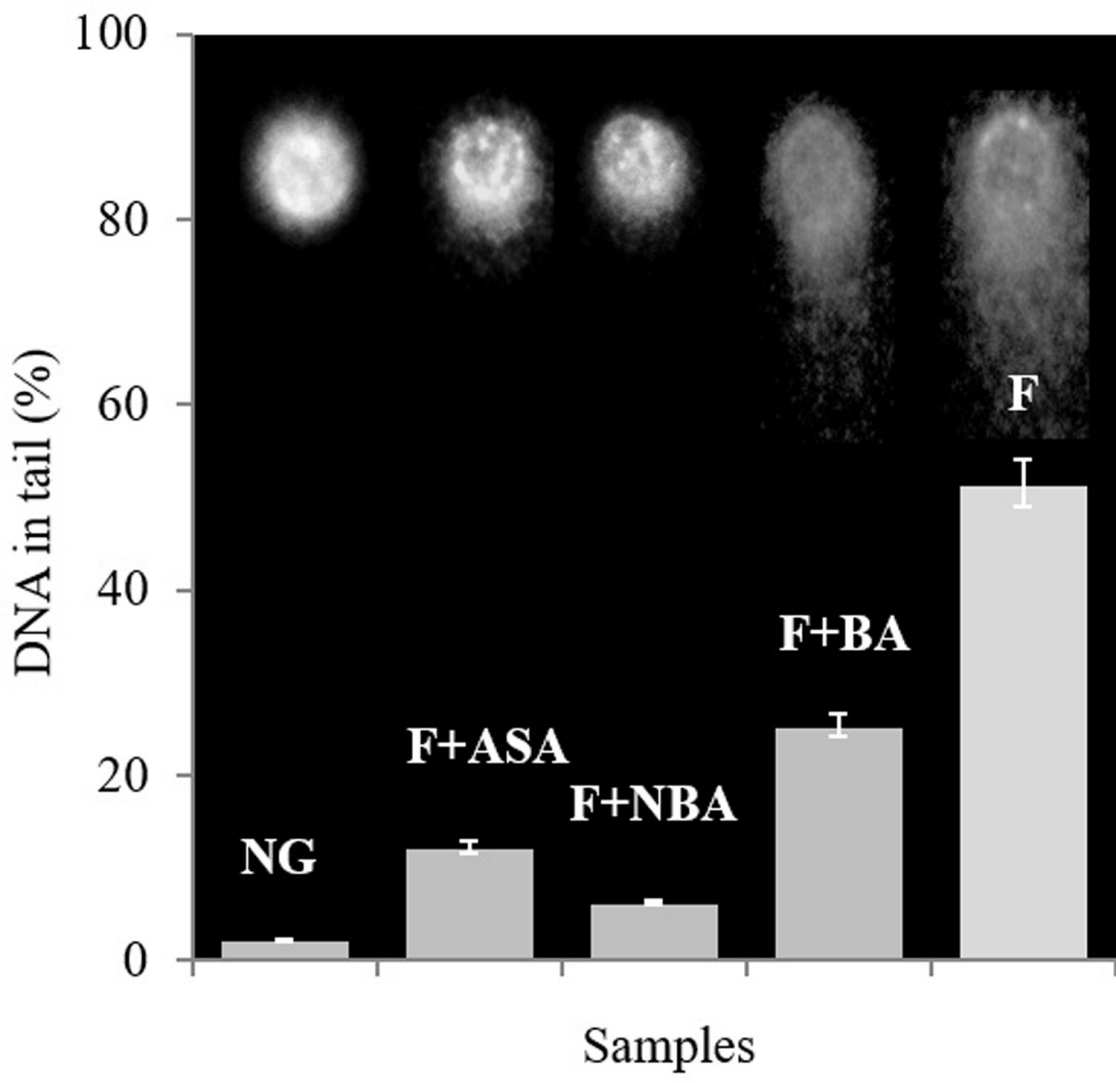
Analogues genetoxocity evaluation. The level of lymphocytes DNA damage presented as photographs and analysed by OpenComet 1.3 imageJ application as mean comet percentage DNA tail in normal human lymphocytes which were treated with non fructatd Hb (NG) and Hb exposed to fructose in the absence (F) or presence of ASA, NBA, and BA after 20 days of incubation. Error bars display the mean ±SD (n = 20).

Comet assay was performed under alkaline state to investigate the impact of fructation in conjunction with the efficacy of ASA, BA, and NBA on averting Hb aggregation and genotoxicity by measuring single- and double-strand breaks in DNA [29]. Fig 5 indicates direct fluorescent microscope images and analyzed data as the percent DNA in the comet tail to compare genotoxicity range on lymphocyte genome after treating with non-fructated Hb and fructated Hb in the absence and presence of chemicals (ASA, BA, and NBA). The significant protective effect against genotoxicity as an indicator of Hb glycation and AGEs production by BA analogues were resulted, which among them treatment by NBA resulted in the highest prevention adequacy on Hb fructation and consequently DNA damage. Moreover, the resulted potency is higher for ASA, than BA treated samples (Fig 5 and S5 Table). All bars resulted from the mean of 20 comets (arbitrary units) analysis for every sample. On the grounds that, reactive oxygen species (ROS) are the primary products of heme degradation and iron releasing stemming from Hb glycation, comet assay was performed to assess the consequence of the genotoxicity caused by ROS release [44]. In this regard, genotoxicity effect of NBA treated samples on lymphocytes genome was about a half and quarter of ASA and BA, respectively which indicates a stronger inhibitory effect of NBA on the glycation process.

**Fig 6:**
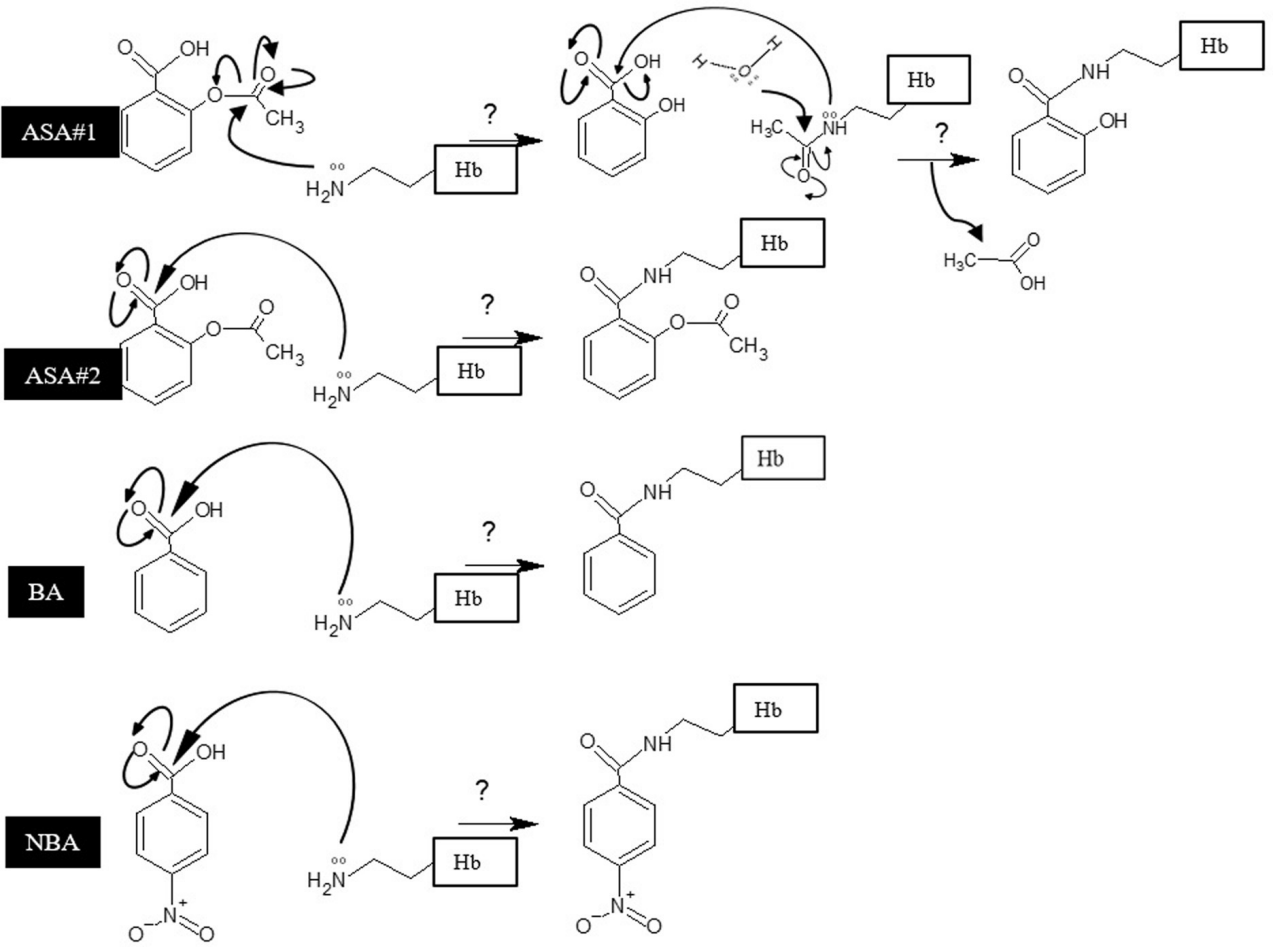
Proposed mechanism of fructation inhibition by ASA, NBA, and BA. ASA#1 represents assumed mechanism for acetylation of Hb free amino groups most likely as N-terminal or lysine residues by ASA followed by benzoyl substitution toward the formation of benzoic acid amide derivative. ASA#2 BA and NBA illustrate nucleophilic attack of free amino groups in protein to carboxyl group from benzoate ring toward the formation of benzoic acid amide derivatives.

Protein glycation is thought to be involved in the development of diabetic complications including neuropathy, nephropathy, retinopathy and cardiovascular diseases [45]. ASA has been shown to acts as an anti-glycating agent [16,21], nevertheless, the exact molecular mechanism whereby ASA exerts its chemopreventive effects is not clearly understood. The acetylation of proteins has been suggested as a major factor involved in some of the unexplained effects of aspirin as well as inhibiting the protein glycation [21,31]. All three chemicals investigated in the present work, regarding their harnessing effect on Hb glycation, possess the fundamental structure as BA. Unlike ASA, two other scrutinized structures; NBA and BA lack the acetyl group. Meanwhile, in the absence of the acetyl group, NBA and BA exhibited more or less adequate inhibitory effects than ASA on Hb fructation, respectively. This report reveals that NBA inhibit various perspectives of glycation with higher potency than ASA. Moreover, based on a study by National Toxicology Program in 1994 on animals during 14 days to 2-year feed study with doses among 40–4900 mg/kg, the non-carcinogenic activity of NBA in low dose and long-term consumption had been admitted [46]. Also, the *in vivo* genotoxicity assay was another proof on nongenotoxicity activities of NBA [46]. However, the prevailing inhibitory effect of NBA on Hb fructation manifests that acetylation could not be the exact mechanism of action of these compounds and other mechanisms as the formation of benzoic acid amide might be involved throughout this process. The possible assumed mechanism involved in witnessed glycation inhibition process is summarized in Fig 6. Admittedly, to explain the antiglycating effect of ASA, acetylation is thought to be a transient step followed by the nucleophilic attack to the carbonyl group in the salicylate to form benzoic acid amide derivatives on Hb. In this regard, mentioned nucleophilic attack in NBA and BA has occurred directly. As a more close report to support our assumption, the reaction of salicylic acid with benzylamine have reported the benzoic acid amide as the only observed product [47]. However, further investigations are required to unravel the exact underlying molecular mechanism entailed their inhibitory effect, moreover reconsidering ASA involved mechanism is emphatically recommended.

## Conclusion

This study provides a contemporary view on the mechanism of action of ASA to support the necessity of further consideration on presumed acetylation mechanism suggested for its protective effect and probably other unexplained curative properties. Moreover, we are introducing NBA as a new glycation inhibitory agent, with LD50 higher than ASA, which needs to be taken into account in the concept of future investigations.

## Supporting information

S1 Table The data related to UV-absorbance at 408 nm for control and glycated samples. (PDF)

S2 Table Data presented the preventive effect of BA analogues on the Hb-AGE production during incubation time by evaluating fluorescence intensity at 370/450 nm (λ ex, λ em). (PDF)

S3 Table Measured ThT emission spectra of samples. ThT emission was recorded by λex 435 nm and λem 505 nm.
(PDF)

S4 Table Table related to samples free amine content percent. (PDF)

S5 Table The percentages of mean DNA in the tail for lymphocyte cells. Lymphocyte genome was exposed to protein samples using comet assay.
(PDF)

## Abbreviations

AGEs: Advanced Glycation End products
Hb: Haemoglobin
RBC: red blood cell
GLUT: glucose transporter
T2DM: type 2 diabetes mellitus
ASA: Acetylsalicyclic acid
NSAIDs: non-steroidal anti-inflammatory drugs
BA: Benzoic acid
NBA: *para*-Nitrobenzoic acid
ThT: thioflavin T
SD: standard deviations
TNBSA: 2,4,6-Trinitrobenzenesulfonic acid assay
SCGE: single cell gel electrophoresis
PBS: phosphate buffered saline
LMPA: Low melting point agarose
NG: non-Glycated
ROS: reactive oxygen species
