# Supplementary material for: Prevention of haemoglobin glycation by acetylsalicylic acid (ASA): A new view on old mechanism
Source: PLoS One. 2019 Apr 15;14(4):e0214725. doi: 10.1371/journal.pone.0214725 (PMC6464172; doi:10.1371/journal.pone.0214725)
Supplement: S1 Table — (PDF) [file pone.0214725.s001.pdf]

S1 Table.

Soret band absorbance at 408 nm for the samples (Fig.1)

Table related to UV-absorbance data at 408 nm

samples \ day	NG	F	F+ASA	F+NBA	F+BA
**0**	0/455	0/455	0/455	0/455	0/455
**3**	0/465666667	0/232	0/267	0/317	0/218
**6**	0/452666667	0/144	0/1885	0/184	0/161
**9**	0/437666667	0/115	0/168	0/191	0/139
**16**	0/447666667	0/106	0/152	0/1895	0/152
**20**	0/450166667	0/11	0/159	0/1905	0/161

Table related to standard deviation of UV-absorbance data at 408 nm

samples \ day	ST-NG	ST-F	ST-F+ASA	ST-F+NBA	ST-F+BA
**0**	0/02275	0/02275	0/02275	0/02275	0/02275
**3**	0/023283333	0/0116	0/01335	0/01585	0/0109
**6**	0/022633333	0/0072	0/009425	0/0092	0/00805
**9**	0/021883333	0/00575	0/0084	0/00955	0/00695
**16**	0/022383333	0/0053	0/0076	0/009475	0/0076
**20**	0/022508333	0/0055	0/00795	0/009525	0/00805

Table related to recorded UV-absorbance at 408 nm for F sample

WL	F day 0	F day 3	F day 6	F day 9	F day 13	F day 16	F day 20
250	0/238666667	0/299	0/321	0/296	0/319	0/317	0/324
252	0/236333333	0/2945	0/318	0/293	0/314	0/313	0/321
254	0/236	0/291	0/3135	0/2915	0/311	0/312	0/32
256	0/235333333	0/2875	0/312	0/29	0/309	0/31	0/319
258	0/236333333	0/2865	0/3115	0/2905	0/308	0/31	0/319
260	0/237	0/2845	0/3105	0/29	0/308	0/31	0/319
262	0/238333333	0/283	0/3075	0/2905	0/308	0/311	0/319
264	0/238666667	0/281	0/3075	0/2905	0/308	0/311	0/319
266	0/239666667	0/2805	0/307	0/2915	0/308	0/31	0/319
268	0/240666667	0/279	0/306	0/2915	0/308	0/31	0/319
270	0/241	0/2775	0/305	0/2915	0/307	0/309	0/317
272	0/241	0/276	0/303	0/291	0/306	0/308	0/316
274	0/241333333	0/275	0/3025	0/291	0/306	0/307	0/314
276	0/240666667	0/273	0/301	0/2905	0/305	0/304	0/312
278	0/24	0/272	0/2995	0/29	0/304	0/302	0/31
280	0/238333333	0/27	0/2975	0/289	0/302	0/3	0/307
282	0/237	0/268	0/295	0/288	0/3	0/296	0/303

284	0/233333333	0/265	0/291	0/285	0/297	0/293	0/299
286	0/227666667	0/259	0/2855	0/281	0/293	0/287	0/292
288	0/223	0/255	0/281	0/2775	0/289	0/282	0/287
290	0/216666667	0/2495	0/2745	0/273	0/284	0/276	0/28
292	0/212666667	0/245	0/2705	0/2695	0/28	0/272	0/275
294	0/204666667	0/2385	0/2635	0/264	0/274	0/265	0/268
296	0/197	0/2305	0/2555	0/257	0/268	0/258	0/26
298	0/192333333	0/2265	0/2505	0/2535	0/263	0/253	0/255
300	0/188333333	0/2225	0/245	0/2485	0/259	0/248	0/25
302	0/184333333	0/2185	0/2405	0/2445	0/254	0/243	0/245
304	0/181333333	0/2155	0/2365	0/2405	0/249	0/239	0/24
306	0/179666667	0/2125	0/234	0/237	0/245	0/235	0/236
308	0/178	0/2105	0/231	0/234	0/242	0/231	0/232
310	0/177666667	0/209	0/228	0/231	0/237	0/228	0/229
312	0/177666667	0/208	0/226	0/2285	0/234	0/226	0/226
314	0/178	0/2065	0/2235	0/226	0/231	0/223	0/224
316	0/178	0/206	0/221	0/2235	0/228	0/221	0/222
318	0/179	0/205	0/2195	0/2215	0/226	0/22	0/22
320	0/18	0/204	0/217	0/219	0/223	0/218	0/218
322	0/181333333	0/203	0/2155	0/217	0/22	0/216	0/217
324	0/182666667	0/202	0/214	0/2145	0/218	0/215	0/215
326	0/185	0/202	0/2115	0/2135	0/216	0/214	0/214
328	0/186333333	0/202	0/2095	0/2115	0/214	0/213	0/213
330	0/188666667	0/201	0/208	0/209	0/211	0/211	0/211
332	0/190666667	0/2005	0/206	0/2065	0/208	0/208	0/208
334	0/192666667	0/2	0/204	0/205	0/206	0/206	0/206
336	0/195666667	0/2	0/203	0/203	0/204	0/204	0/204
338	0/197666667	0/2005	0/2015	0/202	0/202	0/202	0/202
340	0/2	0/2	0/2	0/2	0/2	0/2	0/2
342	0/202333333	0/1995	0/198	0/198	0/197	0/197	0/197
344	0/204666667	0/1995	0/197	0/197	0/196	0/196	0/196
346	0/207333333	0/1995	0/195	0/1955	0/195	0/195	0/194
348	0/210333333	0/1995	0/1945	0/194	0/193	0/194	0/193
350	0/212666667	0/1995	0/1935	0/1935	0/192	0/193	0/192
352	0/215666667	0/1995	0/1925	0/1925	0/191	0/192	0/191
354	0/218	0/1995	0/1915	0/1915	0/19	0/191	0/191
356	0/220666667	0/1995	0/1905	0/1905	0/189	0/19	0/19
358	0/224	0/2005	0/189	0/19	0/188	0/19	0/19
360	0/226333333	0/2005	0/1885	0/1895	0/187	0/189	0/189
362	0/228666667	0/2005	0/187	0/1885	0/186	0/189	0/188
364	0/231666667	0/2005	0/186	0/1885	0/185	0/188	0/187
366	0/233666667	0/2015	0/1855	0/1875	0/184	0/187	0/185
368	0/236333333	0/2015	0/185	0/1865	0/183	0/185	0/183
370	0/24	0/2025	0/1845	0/1855	0/182	0/182	0/18
372	0/244333333	0/2035	0/184	0/1845	0/18	0/179	0/176
374	0/251	0/206	0/1835	0/183	0/178	0/175	0/171
376	0/257	0/208	0/1835	0/182	0/176	0/171	0/167
378	0/264666667	0/2105	0/183	0/181	0/175	0/168	0/164
380	0/273666667	0/2145	0/1835	0/181	0/173	0/166	0/16
382	0/283666667	0/218	0/1835	0/18	0/172	0/163	0/156

384	0/296	0/2225	0/184	0/1795	0/17	0/159	0/152
386	0/308666667	0/2275	0/185	0/179	0/169	0/157	0/149
388	0/318	0/2315	0/1855	0/179	0/169	0/156	0/148
390	0/335333333	0/2385	0/1875	0/179	0/168	0/154	0/145
392	0/355	0/247	0/1885	0/179	0/167	0/152	0/143
394	0/376	0/2555	0/1905	0/18	0/167	0/151	0/141
396	0/401333333	0/266	0/193	0/181	0/166	0/15	0/14
398	0/43	0/2775	0/195	0/1815	0/166	0/149	0/138
400	0/458333333	0/289	0/1985	0/183	0/166	0/148	0/137
402	0/489	0/302	0/2025	0/184	0/167	0/147	0/136
404	0/502	0/308	0/2045	0/1845	0/166	0/146	0/135
406	0/506333333	0/311	0/2055	0/1855	0/166	0/145	0/135
408	0/491333333	0/309	0/2065	0/1855	0/166	0/145	0/134
410	0/471	0/303	0/2055	0/1845	0/165	0/144	0/133
412	0/434333333	0/292	0/2035	0/1835	0/165	0/144	0/132
414	0/387	0/2765	0/1995	0/1815	0/163	0/142	0/13
416	0/355	0/265	0/196	0/179	0/161	0/141	0/129
418	0/312333333	0/2485	0/1905	0/176	0/159	0/139	0/127
420	0/282	0/2355	0/1845	0/1735	0/157	0/138	0/126
422	0/264333333	0/2265	0/181	0/1715	0/156	0/137	0/125
424	0/240333333	0/215	0/1755	0/169	0/154	0/136	0/124
426	0/222666667	0/2055	0/1705	0/1665	0/152	0/135	0/123
428	0/211333333	0/1985	0/167	0/1645	0/15	0/134	0/122
430	0/197	0/1895	0/1625	0/162	0/149	0/132	0/12
432	0/187666667	0/183	0/159	0/16	0/147	0/131	0/119
434	0/181333333	0/179	0/1565	0/1585	0/146	0/13	0/118
436	0/174	0/173	0/153	0/1565	0/144	0/129	0/117
438	0/169666667	0/169	0/151	0/1555	0/143	0/128	0/116
440	0/166	0/166	0/1485	0/154	0/142	0/127	0/115
442	0/162666667	0/163	0/1455	0/1525	0/141	0/127	0/114
444	0/160666667	0/161	0/1445	0/152	0/14	0/126	0/114
446	0/158666667	0/158	0/143	0/1505	0/139	0/125	0/113
448	0/156666667	0/156	0/141	0/1495	0/138	0/125	0/112
450	0/155666667	0/1545	0/1395	0/149	0/137	0/124	0/111
452	0/154333333	0/152	0/1375	0/148	0/136	0/123	0/11
454	0/153333333	0/1505	0/137	0/1475	0/135	0/123	0/11
456	0/152333333	0/1495	0/1355	0/1465	0/135	0/122	0/109
458	0/151333333	0/148	0/1345	0/146	0/134	0/121	0/108
460	0/150333333	0/1465	0/133	0/145	0/133	0/12	0/107
462	0/15	0/1455	0/1315	0/1445	0/133	0/12	0/107
464	0/149333333	0/1445	0/1305	0/144	0/132	0/119	0/106
466	0/148666667	0/1435	0/13	0/143	0/132	0/119	0/106
468	0/147666667	0/1425	0/129	0/1425	0/131	0/118	0/105
470	0/147666667	0/1415	0/128	0/142	0/13	0/118	0/104
472	0/147	0/1405	0/127	0/1415	0/13	0/117	0/104
474	0/147	0/1405	0/1265	0/141	0/129	0/117	0/103
476	0/146666667	0/1395	0/1255	0/1405	0/129	0/116	0/103
478	0/146666667	0/139	0/125	0/14	0/128	0/116	0/102
480	0/146666667	0/1385	0/124	0/1395	0/128	0/115	0/101
482	0/146	0/138	0/124	0/1385	0/128	0/114	0/101

484	0/146333333	0/138	0/1225	0/1385	0/127	0/114	0/1
486	0/147	0/1375	0/1215	0/1385	0/127	0/115	0/101
488	0/147	0/137	0/121	0/1385	0/126	0/114	0/1
490	0/147333333	0/137	0/121	0/138	0/126	0/114	0/1
492	0/147333333	0/137	0/1205	0/1375	0/126	0/114	0/1
494	0/147666667	0/1365	0/1195	0/1375	0/125	0/114	0/099
496	0/147666667	0/136	0/1195	0/1365	0/125	0/113	0/099
498	0/147666667	0/136	0/1185	0/1365	0/125	0/113	0/098
500	0/147333333	0/135	0/1175	0/1365	0/125	0/113	0/098
502	0/147333333	0/135	0/118	0/1365	0/125	0/112	0/098
504	0/147	0/135	0/1175	0/1355	0/124	0/112	0/097
506	0/147	0/1345	0/117	0/1355	0/124	0/112	0/097
508	0/146666667	0/134	0/117	0/1355	0/124	0/111	0/097
510	0/146	0/134	0/116	0/135	0/123	0/111	0/096
512	0/146	0/134	0/116	0/1355	0/123	0/111	0/096
514	0/145666667	0/134	0/1155	0/135	0/123	0/111	0/096
516	0/145	0/1335	0/1155	0/1345	0/123	0/11	0/096
518	0/145	0/1335	0/115	0/1345	0/123	0/11	0/095
520	0/144666667	0/1335	0/1145	0/1345	0/123	0/11	0/095
522	0/144	0/133	0/1145	0/1345	0/122	0/11	0/095
524	0/144	0/133	0/1145	0/1345	0/122	0/109	0/095
526	0/143333333	0/133	0/1145	0/1335	0/122	0/109	0/094
528	0/143	0/1325	0/1135	0/1335	0/122	0/109	0/094
530	0/143666667	0/133	0/113	0/1345	0/122	0/11	0/095
532	0/143333333	0/133	0/1135	0/134	0/122	0/11	0/095
534	0/143	0/1325	0/1125	0/1335	0/121	0/109	0/094
536	0/143	0/133	0/113	0/1335	0/121	0/109	0/094
538	0/142333333	0/132	0/112	0/1335	0/121	0/109	0/094
540	0/141666667	0/132	0/1125	0/133	0/121	0/108	0/093
542	0/141333333	0/1315	0/1115	0/133	0/121	0/108	0/093
544	0/141	0/1315	0/111	0/133	0/121	0/109	0/094
546	0/140333333	0/1315	0/111	0/133	0/121	0/109	0/093
548	0/14	0/131	0/1105	0/1325	0/12	0/109	0/093
550	0/139333333	0/1305	0/11	0/1325	0/12	0/109	0/093
552	0/138333333	0/13	0/11	0/132	0/12	0/108	0/093
554	0/138	0/13	0/1095	0/132	0/12	0/108	0/093
556	0/137333333	0/1295	0/109	0/132	0/12	0/108	0/092
558	0/136666667	0/129	0/109	0/1315	0/12	0/108	0/092
560	0/136666667	0/129	0/1085	0/1315	0/119	0/108	0/092
562	0/136333333	0/1285	0/108	0/1315	0/12	0/108	0/092
564	0/136333333	0/1285	0/108	0/1315	0/12	0/107	0/092
566	0/136333333	0/1285	0/1075	0/1315	0/119	0/107	0/092
568	0/136333333	0/1285	0/107	0/131	0/119	0/107	0/092
570	0/136333333	0/1285	0/1075	0/131	0/119	0/107	0/092
572	0/135666667	0/1285	0/107	0/131	0/119	0/107	0/091
574	0/136	0/1285	0/1065	0/131	0/119	0/107	0/091
576	0/135666667	0/1285	0/106	0/131	0/119	0/107	0/091
578	0/135666667	0/128	0/106	0/131	0/119	0/107	0/091
580	0/135333333	0/1275	0/106	0/131	0/119	0/107	0/091
582	0/135333333	0/1275	0/106	0/1305	0/119	0/107	0/091

584	0/134666667	0/1275	0/1055	0/1305	0/119	0/107	0/091
586	0/134333333	0/127	0/105	0/1305	0/119	0/107	0/091
588	0/134333333	0/1265	0/105	0/1305	0/119	0/107	0/091
590	0/133666667	0/1265	0/1045	0/13	0/118	0/107	0/091
