# Supplementary material for: Prevention of haemoglobin glycation by acetylsalicylic acid (ASA): A new view on old mechanism
Source: PLoS One. 2019 Apr 15;14(4):e0214725. doi: 10.1371/journal.pone.0214725 (PMC6464172; doi:10.1371/journal.pone.0214725)
Supplement: S2 Table — (PDF) [file pone.0214725.s002.pdf]

S2 Table.

Preventive effect of BA analogues on the Hb-AGE production during incubation time by evaluating fluorescence intensity at 370/450 nm (λ_{ex}, λ_{em}) (Fig. 3a)

Table related to AGEs fluorescence emission data					
samples / day	NG	F	F+ASA	F+NBA	F+BA
**0**	63/03333333	67/75	62/06666667	63/56666667	66/13333333
**3**	72/3	131/5	158/3	153/5	164/2
**6**	72/86666667	291/3	234/6	226/4	245/25
**9**	81/33333333	351/65	312/75	289/15	329/6333333
**13**	88/2	412/9	461/2	406/5	521/2
**16**	83/66666667	609/9	560/6	376/1	577/8
**20**	98/16666667	736/4	721/4	603/8	732/05

Table related to standard deviation of AGEs fluorescence emission data					
samples / day	NG	F	F+NBA	F+ASA	F+BA
**0**	3/151666667	3/3875	3/103333333	3/178333333	3/306666667
**3**	3/615	6/575	7/915	7/675	8/21
**6**	3/643333333	14/565	11/73	11/32	12/2625
**9**	4/066666667	17/5825	15/6375	14/4575	16/48166667
**13**	4/41	20/645	23/06	20/325	26/06
**16**	4/183333333	30/495	28/03	18/805	28/89
**20**	4/908333333	36/82	36/07	30/19	36/6025
