# Supplementary material for: Prevention of haemoglobin glycation by acetylsalicylic acid (ASA): A new view on old mechanism
Source: PLoS One. 2019 Apr 15;14(4):e0214725. doi: 10.1371/journal.pone.0214725 (PMC6464172; doi:10.1371/journal.pone.0214725)
Supplement: S3 Table — ThT emission was recorded by λex 435 nm and λem 505 nm. (PDF) [file pone.0214725.s003.pdf]

# S3 Table.

ThT emission spectra of samples, which were recorded by λex 435 nm and λem 505 nm (Fig. 3b)

| Table related to ThT fluorescence emission | | | | | |
|---|---|---|---|---|---|
| **samples / day** | **NG** | **F** | **F+ASA** | **F+NBA** | **F+BA** |
| **0** | 5/2 | 5/2 | 5/2 | 5/2 | 5/2 |
| **6** | 0/1 | 86/35 | 83/333333 | 70/166667 | 89/933333 |
| **9** | 13/733333 | 151 | 93/033333 | 78/066667 | 143/26667 |
| **13** | 7/4 | 272/8 | 240/88333 | 191/61667 | 280/06667 |
| **16** | 12/966667 | 293/65 | 225/7 | 157/91667 | 240/06667 |
| **20** | 13/033333 | 287/2 | 269/26667 | 241/3 | 285/71667 |

| Table related to standard deviation of ThT emission data | | | | | |
|---|---|---|---|---|---|
| **samples / day** | **NG** | **F** | **F+ASA** | **F+NBA** | **F+BA** |
| **0** | 0/26 | 0/26 | 0/26 | 0/26 | 0/26 |
| **6** | 0/005 | 4/3175 | 4/1666667 | 3/5083333 | 4/4966667 |
| **9** | 0/6866667 | 7/55 | 4/6516667 | 3/9033333 | 7/1633333 |
| **13** | 0/37 | 13/64 | 12/044167 | 9/5808333 | 14/003333 |
| **16** | 0/6483333 | 14/6825 | 11/285 | 7/8958333 | 12/003333 |
| **20** | 0/6516667 | 14/36 | 13/463333 | 12/065 | 14/285833 |
