# Supplementary material for: Prevention of haemoglobin glycation by acetylsalicylic acid (ASA): A new view on old mechanism
Source: PLoS One. 2019 Apr 15;14(4):e0214725. doi: 10.1371/journal.pone.0214725 (PMC6464172; doi:10.1371/journal.pone.0214725)
Supplement: S4 Table — (PDF) [file pone.0214725.s004.pdf]

S4 Table.

TNBSA Bar graph (Fig. 4)

Table related to free amine content percent

samples \ day	NG	F	F+ASA	F+NBA	F+BA
**0**	100	100	100	100	100
**3**	101/675978	90/5908096	90	98/2226962	97/6095618
**6**	101/117318	74/9088257	82/9787234	100/103306	99/4023904
**9**	99/2178771	67/1772429	74/6099291	87/1900826	85/059761
**13**	96/7597765	61/487965	74/2553191	90/9090909	71/7131474
**16**	101/675978	66/885485	70/7092199	83/2644628	70/3187251
**20**	101/675978	66/083151	70/7092199	87/2589532	69/749004

Table related to standard deviation of TNBSA data

samples \ day	NG	F	F+ASA	F+NBA	F+BA
**0**	2/01E-14	1/74E-14	1/74E-14	1/74E-14	1/74E-14
**3**	2/03351955	1/81181619	1/8	1/96445392	1/95219124
**6**	2/02234637	1/49817651	1/65957447	2/00206612	1/98804781
**9**	1/98435754	1/34354486	1/49219858	1/74380165	1/70119522
**13**	1/93519553	1/2297593	1/48510638	1/81818182	1/43426295
**16**	2/03351955	1/3377097	1/4141844	1/66528926	1/4063745
**20**	2/03351955	1/32166302	1/4141844	1/74517906	1/39498008
