# Supplementary material for: Prevention of haemoglobin glycation by acetylsalicylic acid (ASA): A new view on old mechanism
Source: PLoS One. 2019 Apr 15;14(4):e0214725. doi: 10.1371/journal.pone.0214725 (PMC6464172; doi:10.1371/journal.pone.0214725)
Supplement: S5 Table — Lymphocyte genome was exposed to protein samples using comet assay. (PDF) [file pone.0214725.s005.pdf]

S5 Table.

Mean DNA in tail percentages for lymphocyte cells, which were exposed to protein samples (Fig 5)

Samples	Tail DNA%	Standard Deviation
**NG**	2/265990585	2/265990585
**F+ASA**	12/26054518	12/26054518
**F+NBA**	6/284644203	6/284644203
**F+BA**	25/37763659	25/37763659
**F**	51/57183899	51/57183899
